# Visual mislocalization during double-step saccades

**DOI:** 10.3389/fnsys.2015.00132

**Published:** 2015-10-19

**Authors:** Eckart Zimmermann

**Affiliations:** Neuroscience Institute, National Research CouncilPisa, Italy

**Keywords:** saccade, compression, mislocalization, double-step saccade, visual space perception

## Abstract

Visual objects presented briefly at the time of saccade onset appear compressed toward the saccade target. Compression strength depends on the presentation of a visual saccade target signal and is strongly reduced during the second saccade of a double-step saccade sequence (Zimmermann et al., 2014b). Here, I tested whether perisaccadic compression is linked to saccade planning by contrasting two double-step paradigms. In the same-direction double-step paradigm, subjects were required to perform two rightward 10° saccades successively. At various times around execution of the saccade sequence a probe dot was briefly flashed. Subjects had to localize the position of the probe dot after they had completed both saccades. I found compression of visual space only at the time of the first but not at the time of the second saccade. In the reverse-direction paradigm, subjects performed first a rightward 10° saccade followed by a leftward 10° saccade back to initial fixation. In this paradigm compression was found in similar magnitude during both saccades. Analysis of the saccade parameters did not reveal indications of saccade sequence preplanning in this paradigm. I therefore conclude that saccade planning, rather than saccade execution factors, is involved in perisaccadic compression.

## Introduction

Saccade eye movements displace the eyes with high-speed motion around three times per second, implying the need of a counteracting mechanism which stabilizes the perception of visual space. Saccades cause massive perceptual distortions: stimuli flashed briefly around the time of saccades are mislocalized in the direction of the saccade (Ross et al., 2001) and compressed towards the saccade target (Morrone et al., 1997; Ross et al., 1997; Lappe et al., 2000; Zirnsak and Moore, 2014; Zirnsak et al., 2014). We have recently shown that perisaccadic compression of space is strongly reduced if saccades are performed into the void without the presentation of a visual saccade target signal (Zimmermann et al., 2014c). This is consistent with the idea that saccadic compression is the signature of a mechanism attempting to match objects seen before the saccade with those seen after Cicchini et al. (2013) and Zimmermann et al. (2014a).

Perisaccadic compression has also been tested in a double-step saccade paradigm (Lavergne et al., 2012; Zimmermann et al., 2014b). In this paradigm—which had been introduced by Westheimer (1954) and Hallett and Lightstone (1976)—two saccade targets are shown, to which subjects saccade sequentially. Usually both targets are switched off before the first saccade of the sequence is initiated so that the second saccade is guided by the memorized position of the targets (while the first can be programmed as a simple sensory-to-motor transformation). Under these conditions we found compression only at the time of the first but not at the time of the second saccade. However, when we presented the saccade targets for 500 ms and delayed saccade execution, we found compression during both saccades. We interpreted this result as evidence that compression is linked to saccade planning. In saccade sequences with short saccade target presentation durations both saccades might be preplanned in parallel (Becker and Jüergens, 1979; McPeek et al., 2000). The temporal interval between the first and the second saccade in a double step paradigm can be as short as 20 ms, far less than the 125 ms (minimum) required for programming of single saccades (Becker and Jüergens, 1979). Other evidence for saccade sequence coding comes from a study which showed that saccade parameters like latency and duration change with the length of the saccade sequence (Zingale and Kowler, 1987). Saccade preplanning is further supported by the inverse relationship between the latency of the first saccade, and the intersaccadic interval between the two (Becker and Jüergens, 1979; McPeek et al., 2000). However, when saccade targets are presented long and the sensorimotor system has time to encode them in spatiotopic coordinates (Sharika et al., 2014; Zimmermann et al., 2014d), the visual saccade target signal which drives perisaccadic compression is available in both saccades again.

Here, I sought to further test the idea of a connection between saccade pre-planning and compression. I contrasted perisaccadic compression in two double-step paradigms: in a paradigm, where two saccades had to be performed consecutively in the same direction, the analysis of saccade parameters suggested that both saccades were preplanned in parallel before execution of the first saccade. However, in a reverse-direction paradigm, where the second saccade had to go back to the initial fixation position, no indications for preplanning were found in the analysis of saccade parameters. If perisaccadic compression is linked to saccade planning, then the absence of compression at the time of the second saccade should only be found in the same-direction double-step paradigm. In the reverse-direction paradigm however, where saccades are planned sequentially, compression should occur during both saccades.

## Materials and Methods

### Participants

Three subjects (one author, two naive subjects, mean *age* = 29 years) participated in all experiments. All subjects had normal or corrected-to-normal vision. Subjects gave informed consent. Experimental procedures were approved by the local ethics committee [Comitato Etico Pediatrico Regionale-Azienda Ospedaliero-Universitaria Meyer-Firenze (FI)] and are in line with the declaration of Helsinki.

### Apparatus

The subject was seated 57 cm from a 22″ CRT color monitor (Barco Calibrator) with head stabilized by chin- and head-rest. The visible visual field was 40 × 30°. Stimuli were presented on the monitor with a vertical frequency of 120 Hz at a resolution of 800 × 600 pixels. Eye movements were monitored by the Eyelink 1000 system (SR Research, Ltd., Canada), which samples gaze positions with a frequency of 2000 Hz. Viewing was binocular but only the dominant eye was recorded. The system detected start and end of a saccade when eye velocity exceeded or fell below 22 deg/sec and acceleration was above or below 4000°/s^2^. In all experiments the background was red (7 cd/m^2^) and the fixation points and saccade targets were black (0.5 cd/m^2^).

### Same-Direction and Reverse-Direction Double Step Paradigm

**Figure 1:**
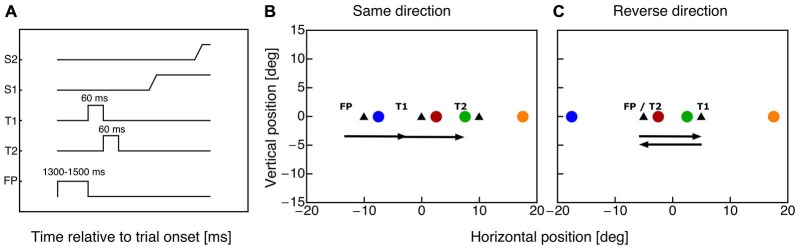
**(A)** Timecourse of events in the double step task. The fixation point was shown for a variable time between 1300 and 1500 ms. With offset of the fixation point the first saccade target (T1) appeared for 60 ms. Simultaneously with the disappearance of target T1 saccade target T2 was presented for 60 ms. The subject performed two horizontal saccades (S1 and S2) to the remembered target positions. **(B)** Positions of fixation point and saccade targets (triangle symbols) in the same-direction double-step paradigm. The colored dots indicate the eight possible probe dot locations. Horizontal probe positions were: −7.5°, 2.5°, 7.5° and 17.5°. **(C)** Positions of fixation point and saccade targets (triangle symbols) in the cross-direction double-step paradigm. The colored dots indicate the eight possible probe dot locations. Horizontal probe positions were: −17.5°, −2.5°, 2.5° and 17.5°.

Subjects fixated a fixation point for 1000 ms plus a random duration between 300–500 ms (see Figure 1A). Then, the fixation point was switched off and the first saccade target appeared for 60 ms. With offset of the first saccade target the second saccade target was shown for 60 ms. In the same-direction double-step sessions subjects were required to execute two 10° saccades in rightward direction (see Figure 1B). In the reverse-direction double step sessions subjects had to perform two 10° horizontal saccades, the first rightward and the second leftward back to the fixation point (see Figure 1C). Subjects were instructed to initiate the saccade sequence as soon as the first target appeared. Since the saccade reaction time was around 160 ms, both saccadic targets had been disappeared before the first saccade starts. A probe dot (diameter: 0.75°, green: 18.6 cd/m^2^) was presented for 10 ms at a time randomly chosen in one of four positions (see Figures 1B,C). The probe positions were chosen such that within each paradigm two probes were centered around saccade target T1 and two probes around saccade target T2. Both probe pairs had identical distances to the specific saccade target. All probes were shown on the horizontal meridian at the following horizontal positions for the same-direction paradigm: −7.5°, 2.5°, 7.5° and 17.5°, for the reverse-direction paradigm: −17.5°, −2.5°, 2.5° and 17.5°. A mouse cursor appeared 1000 ms after the offset of the second saccade target, which the subject had to use to indicate the apparent position of the probe dot.

### Compression Index

To quantify the magnitude of perisaccadic mislocalization, a compression index was calculated. Perceived probe positions were plotted against physical probe positions and the slope of the regression was taken to indicate compression magnitude across all four probe positions. If localization is veridical, the average data should fall on the equality line, with a slope of 1. If probe stimuli on average are compressed, all stimuli will be seen at the same position, so the slope of the best-fitting regression will be zero. The slope of the regression subtracted from 1 served as the index for compression magnitude. The higher this index is, the higher the amount of compression.

## Results

### Saccade Parameters

**Figure 2:**
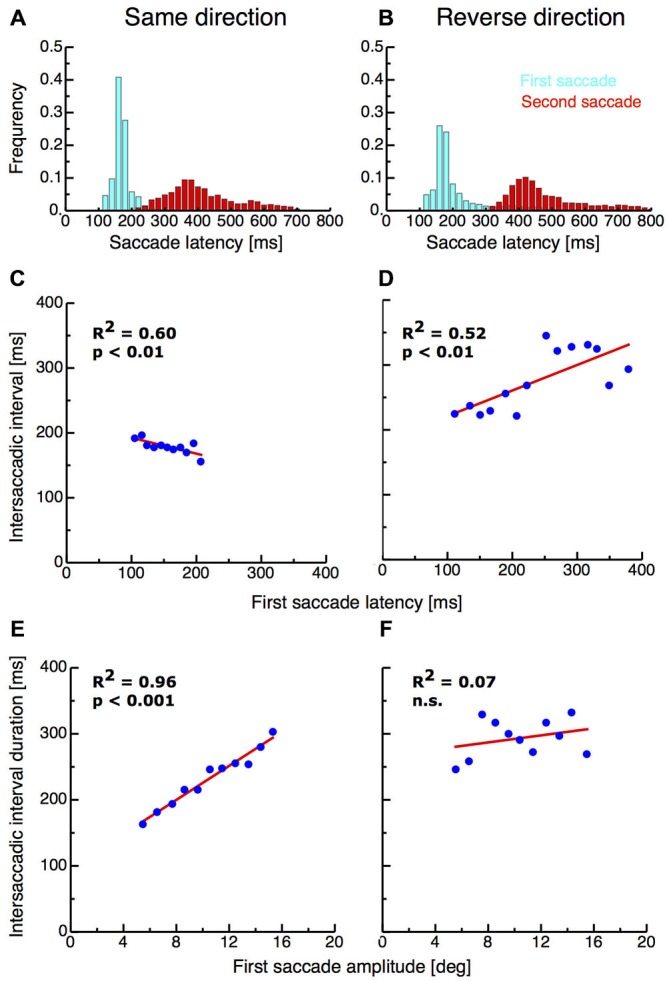
**(A,B)** Saccade latencies for the first saccades and the intersaccadic interval in the same-direction and the reverse double-step task. Data are binned into bins with a width of 20 ms. **(C,D)** Intersaccadic interval duration as a function of first saccade latency in the same-direction double-step task. **(E,F)** First saccade amplitude as a function of intersaccadic interval duration.

I first analyzed the saccade parameters of the first and the second saccades in both double-step paradigms. Previous studies have found that the interval between end of the first and start of the second saccade in the double-step paradigm can be extremely short and suggested that the second saccade must therefor be preplanned before execution of the first (Becker and Jüergens, 1979). Figures 2A,B shows the latency distribution of first (shown in cyan) and second saccades (shown in red) in the same-direction and the reverse-direction paradigm. The mean latency of first saccades in the same-direction paradigm was 159.99 ms (SD 27.02 ms) whereas on average latencies in the reverse-direction paradigm were longer with a mean of 200.80 ms (SD 90.11 ms). First saccade latencies in the same-direction paradigm were significantly shorter than in the reverse-direction paradigm (ttest, *p* < 0.001). The intersaccadic interval duration in the same-direction paradigm ranged from 21—937 ms with a mean duration of 190.71 ms (SD 111.62 ms). The occurrence of saccades with these extreme short latencies indicates that the paradigm induced pre-planning of the two-saccade sequence. Importantly, the reverse-direction paradigm generated higher intersaccadic intervals, ranging from 52–1337 ms with a mean of 248.53 ms (SD 119.16 ms). Intersaccadic interval durations were significantly higher in the reverse-direction than in the same-direction paradigm (t-test, *p* > 0.001).

If the second saccade is preplanned before execution of the first saccade, then longer first saccade latencies should imply shorter intersaccadic interval durations since most of the saccade planning is already terminated. I found a significant negative correlation (*R*^2^ = 0.6, *p* < 0.01) between first saccade latencies and intersaccadic interval duration in the same-direction paradigm, confirming the results of earlier studies (Becker and Jüergens, 1979; McPeek et al., 2000; see Figure 2C). By contrast however, a significant positive correlation (*R*^2^ = 0.52, *p* < 0.01) between first saccade latencies and intersaccadic interval duration was observed in the reverse-direction paradigm (see Figure 2D). This result suggests that in this paradigm, saccades were planned independently from each other.

As another indicator for saccade sequence preplanning, it has been reported that hypometric first saccades were almost always followed by very short latency second saccades (McPeek et al., 2000). Indeed, I found a very strong correlation between the size of the first saccade amplitude and the duration of the intersaccadic interval (*R*^2^ = 0.96, *p* < 0.001; see Figure 2E). In the reverse-direction paradigm however no correlation was observed (*R*^2^ = 0.07, n.s.; see Figure 2F).

### Mislocalization

**Figure 3:**
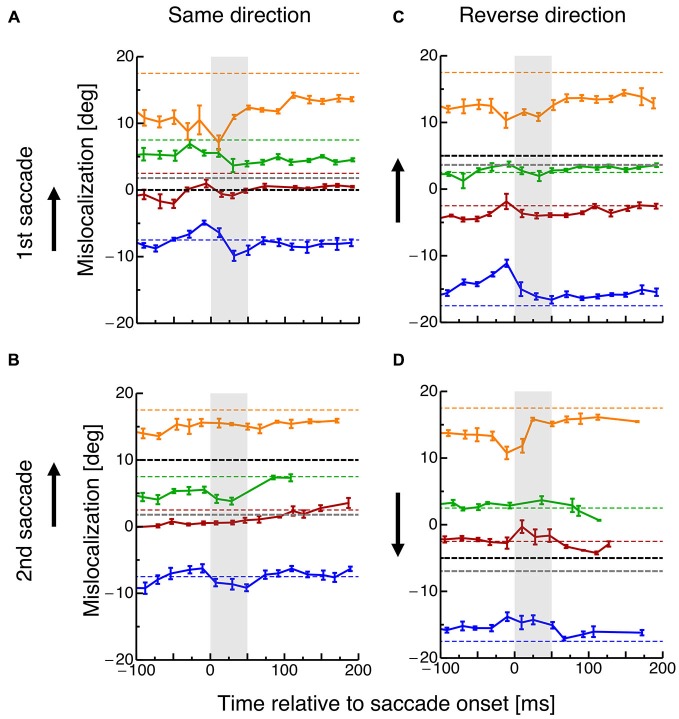
**(A)** Localization of the probe dot relative to onset of the first saccade in the same-direction double-step task. Different colors refer to the eight different positions of the probe dot as shown in Figure 1B. Data are pooled across subjects and binned into bins with a width of 20 ms. Error bars represent S.E.M. **(B)** Localization of the probe dot relative to onset of the second saccade in the same-direction double-step task. Same conventions as in **(A)**. **(C)** Localization of the probe dot relative to onset of the first saccade in the reverse-direction double-step task. Same conventions as in **(A)**. **(D)** Localization of the probe dot relative to onset of the second saccade in the reverse-direction double-step task. Same conventions as in **(A)**.

In order to investigate how the planning of saccade sequences affects visual space, subjects had to localize the spatial position of briefly flashed objects. Figure 3 shows mislocalization for four different probe dot locations tested at various times around execution of the two saccades. The black dotted line indicates the position of the saccade target and the gray dotted line the average saccade landing position. Data are pooled across all subjects and binned into bins with a width of 20 ms. In order to check whether a significantly different amount of trials entered the perisaccadic bins in the different conditions, a 2 × 2 ANOVA was calculated across paradigms (same-direction/reverse-direction) and saccade number (1st/2nd). No significant differences were revealed.

**Figure 4:**
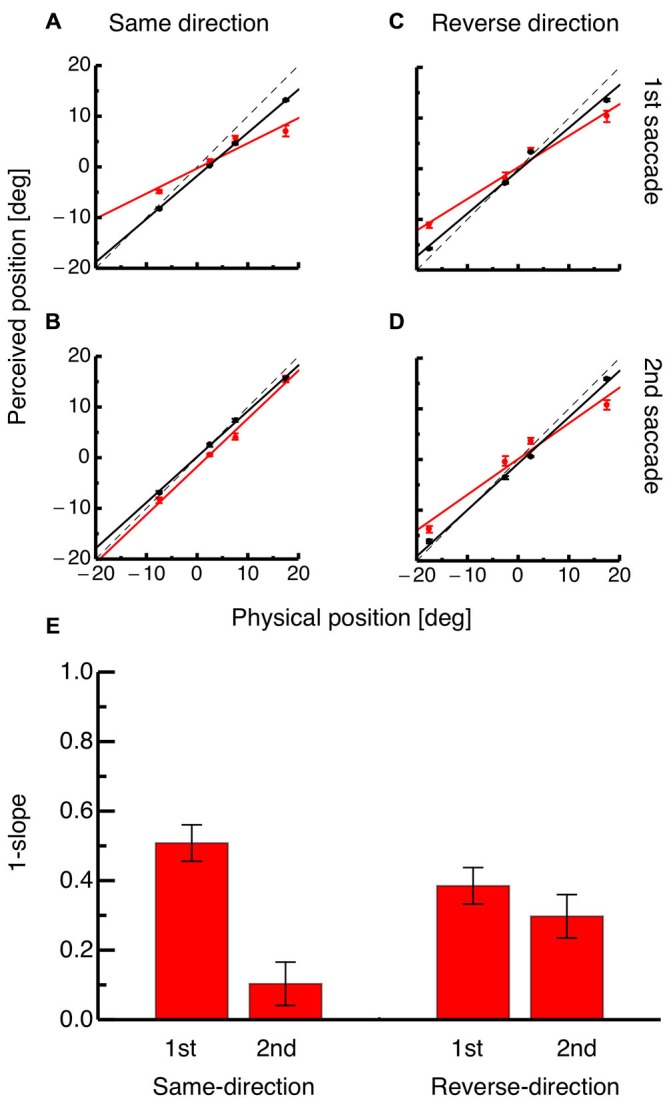
**(A)** Physical position of the probe dot against perceived position for dots presented immediately at onset of the first saccade (shown in red) and for dots presented at least 100 ms after onset of the first saccade (shown in black) in the same-direction double step paradigm. Lines represent the linear fit of the data (trials averaged across subjects) and error bars represent S.E.M. **(B)** Physical position of the probe dot against perceived position for dots presented immediately at onset of the second saccade (shown in red) and for dots presented at least 100 ms after onset of the second saccade (shown in black) in the reverse-direction double step paradigm. Same conventions as in **(A)**. **(C)** Physical position of the probe dot against perceived position for dots presented immediately at onset of the first saccade (shown in red) and for dots presented at least 100 ms after onset of the first saccade (shown in black) in the reverse-direction double step paradigm. Same conventions as in **(A)**. **(D)** Physical position of the probe dot against perceived position for dots presented immediately at onset of the second saccade (shown in red) and for dots presented at least 100 ms after onset of the second saccade (shown in black) in the reverse-direction double step paradigm. Same conventions as in **(A)**. **(E)** Average compression indices, reflecting the slope of the regression through average probe localization at the time of saccade onset. Error bars were derived by bootstrapping.

To quantify the perisaccadic mislocalization strength, compression indices were calculated for both saccades in both paradigms as shown in Figures 4A–D. Each panel shows average perceived position for each of the four probe dots against their physical positions at either the time of the saccade (shown in red) or 100 ms after it (shown in black). For the same-direction paradigm, the occurrence of compression at the time of the first saccade is indicated by the slope of the regression (shown in red in Figure 4A). However, at the time of the second saccade the slope of the regression for average data from the perisaccadic range (shown in red) is almost identical to the slope of the regression for probe dots presented after saccade initiation (see Figure 4B). In the reverse-direction paradigm, compression was seen at the time of both, the first and the second saccade (see Figures 4C,D).

Figure 4E shows average compression indices derived from bootstrapping. A bootstrap signed ttest was performed between mislocalization at the time of the first and the second saccade for the same-direction as well as the reverse-direction paradigm. A significant difference in mislocalization magnitude was found in the same-direction paradigm (*p* < 0.01) but not in the reverse-direction paradigm. The difference in compression strength between the first and the second saccade was significantly higher in the same-direction than in the reverse-direction paradigm (*p* = 0.008).

## Discussion

I found that in a double-step paradigm where two sequential saccades were performed in the same direction, perisaccadic compression of visual space only occurred during the first but not during the second saccade. However, in a paradigm where the second saccade went into the reverse direction of the first, compression had the same magnitude during both saccades. The two saccade targets were successively flashed, each lasting 60 ms. Since the average saccade latency was 159 ms, planning of the two saccades could not rely on the visual saccade target signal but required a memory representation of it. Several studies suggest that the oculomotor system pre-plans both saccades in advance in this double-step paradigm (Becker and Jüergens, 1979; McPeek et al., 2000).

I contrasted two double-step paradigms which differed in how much they allowed saccade sequence preplanning. Indications of saccade sequence pre-planning were either absent or reduced in the reverse direction paradigm. The idea of preplanning arose because intersaccadic interval durations in a two-saccade sequence can be as low as 20 ms (Becker and Jüergens, 1979). I found short intervals of ~20 ms only in the same-direction but not in the reverse-direction paradigm. Evidence for saccade sequence preplanning has been also observed in an inverse relationship between first saccade latency and the intersaccadic interval duration (Becker and Jüergens, 1979; McPeek et al., 2000). I found this negative correlation only in the same-direction but not in the reverse-direction paradigm. A third indicator for preplanning is a correlation between primary saccade amplitude size and intersaccadic interval duration. It had been suggested that hypometricity in the first saccade of the sequence occurs because the amplitude planning of that saccade is disturbed by the concurrent planning of the second saccade (McPeek et al., 2000). Thus, if the intersaccadic interval duration is short, much of second saccade planning took place in parallel to first saccade planning, resulting in a stronger hypometricity. Again, I found a significant correlation only in the same-direction paradigm.

How does preplanning explain the absence of compression at the time of the second saccade? We have shown previously that saccades which are performed “into the void” without the presentation of a saccade target do not induce perisaccadic compression (Zimmermann et al., 2014c). Similarly, Luo et al. (2014) observed reduced compression magnitudes when no saccade target was presented. We also observed absence of compression during the second saccade of a double-step saccade sequence, where the first saccade was in vertical and the second saccade in horizontal direction (Zimmermann et al., 2014b). We have argued that the visual signals of the saccade targets are stored only in retinotopic coordinates when presented in that very short manner. Thus, at the time of the second saccade no target signal was available, neither in retinotopic nor in spatiotopic coordinates. Since the functional role of compression is to integrate corresponding objects, i.e., the probe dot and the saccade target, in space across saccades (Cicchini et al., 2013; Zimmermann et al., 2014a), no compression is seen during the second saccade. When we presented each saccade target for 500 ms, compression was observed during both saccades. In that condition spatiotopic saccade target signal might have become available, consistent with earlier findings that spatiotopic representations take time to build up Zimmermann et al. (2014d). However, in the same-direction paradigm, which was tested here, both saccades are of the same size and direction. Thus, after execution of the first saccade the spatiotopic position of the second saccade target position coincides with the retinotoipic position of the first saccade target. The absence of compression during the second saccade of the same-direction paradigm therefore is unlikely to be related to a lack of updating of the visual saccade target signal. Even if the second saccade target is not updated after the execution of the first saccade, the retinotopic position of the first saccade target could serve as the reference.

Saccade preplanning might also change other factors like perisaccadic suppression (Volkmann, 1986; Diamond et al., 2000) which then influence compression. However, how suppression is modulated during double-step saccades has not yet been investigated.

I conclude that perisaccadic compression of visual space is generated by factors associated to saccade planning and not to saccade execution.

## Conflict of Interest Statement

The Reviewer Guido Marco Cicchini declares that, despite being affiliated to the same institution as the author Eckart Zimmermann, the review process was handled objectively and no conflict of interest exists. The author declares that the research was conducted in the absence of any commercial or financial relationships that could be construed as a potential conflict of interest.
